# A preliminary study on the neurocognitive deficits associated with loneliness in young adults

**DOI:** 10.3389/fpubh.2024.1371063

**Published:** 2024-04-12

**Authors:** Eunju Jin, Samuel Suk-Hyun Hwang

**Affiliations:** Department of Psychology, Chonnam National University, Gwangju, Republic of Korea

**Keywords:** loneliness, neurocognitive functioning, executive function, attention, depression, young adults

## Abstract

The experience of loneliness is universal and may have an adverse effect on neurocognitive functioning even at a younger age. Using a comprehensive neurocognitive functioning test (NCFT) battery, we examined the possible negative effects of loneliness on neurocognitive functioning in young adults. The high-loneliness and low-loneliness groups were screened using the UCLA Loneliness Scale v. 3, and measures pertaining to the domains of intelligence, attention, memory, executive function, and psychomotor functioning were tested and compared. As depression and anxiety were significantly higher in the high-loneliness group, an analysis of covariance was conducted. As a result, the high-loneliness group showed significantly poor performance on measures of executive function and attention prior to controlling for depression and anxiety, and executive function retained its significance even after controlling for these variables. Additional analysis showed that depression and anxiety did not significantly mediate the relationship between loneliness and neurocognitive functioning. Such results suggest that loneliness is likely to negatively affect executive functioning and attention in early adulthood and then progressively spread to other domains of cognitive functioning, as reported in the older adult population. The limitations and implications of the present study were considered and addressed.

## Introduction

Positive emotional exchange with others is a source of happiness for most people. However, when this exchange does not sufficiently meet our needs and expectations, we often feel frustrated and lonely. In some cases, loneliness can accompany physiological changes and even somatic symptoms associated with depression (1). However, the impact of loneliness may be more profound, as past studies on the mostly older adult population have consistently reported that its negative effect may even extend to neurocognitive functioning (2). Vulnerability toward loneliness, however, is universal and exposure to chronic loneliness may have an adverse effect on neurocognitive functioning even at a younger age. Accordingly, we examined the possible negative effects of loneliness on neurocognitive functioning in the young adult population.

Loneliness has been found to be a significant risk factor for cognitive decline, such that the severity of loneliness was found to be inversely related to performance on cognitive tests (3). In a prospective study on the older adult population, those with high levels of loneliness showed significantly higher cognitive deficits compared with those with low levels of loneliness (4). Similarly, both chronic and transient loneliness were predictive of the negative consequences for cognitive functioning and the health of the brain in the older adult population (5). The cognitive domains adversely affected by loneliness in the older adult population included memory, attention, language, and executive function (6, 7). Some inconsistencies in the results, nonetheless, are present such that some studies [e.g., (8)] have reported a bidirectional relationship between loneliness and cognition, while a prospective study by McHugh Power et al. (9) found that attention may affect loneliness but not vice versa. A recent meta-analysis of older adults without dementia (10) found that loneliness was associated with poorer global cognition, episodic memory, working memory, visuospatial function, processing speed, and semantic verbal fluency.

As described above, most past studies on the association between loneliness and neurocognitive functioning deficits have focused mostly on the older adult population. For example, a recent systematic review (11) that examined “the impact of social isolation and loneliness on memory in middle- and older-aged adults” in PubMed, Scopus, and PsycINFO databases until January 2022 found 11 studies whose minimum age of participants was 50 years and only 1 study with the age of the participants being ≥45 years. More recently, a few studies that extended their investigations to include middle-aged populations in their 40s have reported significant findings on the relationship between loneliness and cognitive functioning (12–16). Specifically, loneliness was linked with impairments in executive functioning (16) and memory (12, 13, 15, 16) but not in global cognition, verbal learning, and fluency (12). In this population, persistent loneliness has been found to be associated with smaller parietal, temporal, and hippocampus volumes, which are responsible for memory and executive dysfunction (16). In addition, a higher level of education has been identified as the mediating factor (12, 15) supporting the view that cognitive reserve may serve as a protective factor (17).

In contrast, the effect of loneliness on the neurocognitive functioning of the young adult population has not been extensively examined, even though this age group may be particularly vulnerable to loneliness (18). Loneliness in the younger population is likely to show significant relationships with a narrow band of deficits in neurocognitive functioning compared with the middle-aged counterpart because of the progressive nature of the deficits in cognitive functions (19). A study based on college students has reported the negative effect of loneliness on their social cognition, which caused biased information processing about social relationships and their outcomes (20). It is, however, unclear whether loneliness holds implications for other cognitive functions as found in their middle-aged counterparts.

The negative effect of depression has been examined more extensively in the younger population and may provide some insights since loneliness has been closely linked with depression (21). In general, the domains of attention, verbal memory, visual memory, verbal reasoning/knowledge, and IQ were found to be affected by depression (22). In a recent longitudinal study, depression and anger symptoms were found to be associated with declines in episodic memory and executive functioning (23). Such cognitive domains should be more vulnerable to the adverse effects of loneliness than others in this population, although the relationship between loneliness and depression is likely to be bidirectional (24). However, it should also be mentioned here that some studies have demonstrated that loneliness does not always lead to depression. For example, variables such as self-disgust have been identified to mediate between loneliness, depression, and anxiety (25), and positive coping styles have also been found to alleviate the effect of loneliness on depression (26, 27). Accordingly, in order to delineate the pure effect of loneliness on neurocognitive functioning in a young population, it may be essential to address the effect of depression and anxiety, which may mediate between loneliness and performance on neurocognitive functioning test (NCFT) battery (28, 29).

We, therefore, conducted a preliminary study on the effect of chronic loneliness in a university student population using a comprehensive neurocognitive functioning test battery, which included measures of general IQ, memory, attention, executive functioning, and psychomotor speed. These cognitive domains largely overlap those suggested by the American Psychiatric Association (30) to be considered when assessing cognitive functioning in mental disorders. And since the performance on these cognitive tasks is invariably affected by the emotional state of the subjects (31), we have controlled for depression and anxiety using an ANCOVA. In addition, we also carried out a *post-hoc* mediational analysis to examine the possible influence of those variables on the association between loneliness and cognitive deficits.

## Methods

### Participants

The study was conducted on an initial pool of 365 undergraduate students residing in Gwangju, Korea. The participants completed the initial survey, which included demographic information and psychological scales, including Russell’s UCLA Loneliness Scale v. 3 [RULS v.3: (32, 33)]. Then, 2 months later, they were asked to complete RULS v.3 again. Only those who scored in either the highest or lowest quartiles of this scale at both times were asked to participate in additional NCFT, whereby three people were excluded because they were no longer in the top quartile. As a result, 33 (male = 45%) out of 99 (male = 41%, age = 20.90, SD = 2.29; RULS v.3 mean = 22.09, SD = 11.39, range 4 ~ 24) participants in the lowest quartile and 21 (male = 35%) out of 101 (male = 40%, age = 21.16, SD = 2.66; RULS v.3 mean = 38.24, SD = 7.52, range 27 ~ 57) participants in the highest quartile at the second measurement phase agreed to further procedure, respectively. There was no significant difference in terms of age, education, and gender ratio (*χ*^2^ = 0.28, n.s.) between the highest- and the lowest-quartile groups who took the NCFT (see Table 1). As a note, in the lowest quartile, there was no significant difference in the RULS v.3 score between those who agreed and those who refused to participate in the NCF testing (*t* = 1.66, n.s.), but in the highest quartile, those who agreed to NCF testing had a significantly lower RULS v.3 score than those who refused (*t* = 2.53*). There was no significant difference between those who agreed and those who refused to participate in age for both the highest quartile (*t* = −0.12, n.s.) and the lowest quartile (*t* = 0.83, n.s.), respectively. None of the participants reported being under treatment or medication for any psychiatric problems, and all were right-handed.

**Table 1:** Group differences in Neuro-cognitive measures according to independent groups *t*-test or Mann-Whitney U test.

Variables		Possible Range	Cronbach’s *α*	Low-Loneliness M(SD)	High-Loneliness M(SD)	*t or U*
Age (yrs.)				20.90 (2.29)	21.09 (2.18)	−0.29
Education (yrs.)				12.42 (0.79)	12.52 (0.98)	−0.41
Loneliness		0~60	*0.93*	15.58 (6.20)	35.38 (4.61)	−18.70***
Depression		0 ∼ 60	*0.91*	10.27 (7.17)	24.67 (13.06)	−4.63**
Anxiety		20 ∼ 80	*0.93*	27.12 (8.56)	37.85 (8.56)	−4.56**
IQ	K-WAIS Vocabulary	0 ∼ 57	*0.65* *0.65*	39.97 (3.80)	40.10 (3.80)	−0.12
	Block design	0 ∼ 66	*0.93*	55.70 (8.40)	53.00 (11.21)	1.09
Attention	K-WAIS Digit span forward	0 ∼ 16	*0.85*	13.67 (1.65)	12.52 (1.75)	2.42*
	Digit symbol-coding	0 ∼ 135	*-*	98.33 (17.15)	93.43 (14.85)	−1.50
Memory	ROCF Copy	0 ∼ 36	*0.64* *–*	35.54 (0.65)	35.64 (0.59)	−0.91
	Immediate	0 ∼ 36	*–*	27.89 (4.65)	26.50 (4.07)	−1.48
	Delayed	0 ∼ 36	*–*	27.40 (5.05)	26.16 (3.72)	−1.71
	AVLT Immediate recall error	–	*0.77* *–*	0.18 (0.58)	0.23 (0.43)	−1.63
	Delayed recognition error	–	*–*	0.12 (0.33)	0.19 (0.51)	−0.18
Executive function	WCST Perseveration	–	*–*	41.06 (7.56)	46.33 (9.00)	−2.32*
	STROOP Word error	–	*0.70*	0.06 (0.24)	0.19 (0.40)	−1.36
	Color-word error	–		0.18 (0.53)	0.24 (0.54)	−0.51
	Color-nonword error	–		0.15 (0.44)	0.62 (0.74)	−2.69**
	Color-word mismatch error	–		0.36 (0.70)	1.05 (1.07)	−2.32*
	Interference error			0.30 (0.73)	0.86 (1.12)	−1.94^†^
Psycho-Motor	TMT A trial error	–	*0.38*	0.09 (0.29)	0.00 (0.00)	1.79
	B trial error	–		0.15 (0.44)	0.24 (0.54)	−0.57

*p < 0.05, **p < 0.01, ***p < 0.001, ^†^*p* < 0.07.AVLT, Auditory Verbal Learning Test; IQ, General Intelligence; K-WAIS, Korean-Wechsler Intelligence Scale; ROCF, Rey-Osterrieth Complex Figure Drawing Test; TMT, Trail Making Test; WCST, Wisconsin Card Sorting Test.

### Procedure

This study was conducted with the approval of the research ethics committee of Chonnam National University (IRB No.: 1040198-160422-HR-027-03), and all procedures were administered after the participants had signed the written informed consent form.

Each participant first completed self-reported measures of depression and anxiety and subjective loneliness for a preliminary validation study on the RULS v.3 (32, 33). For the measure of depression, the Center for Epidemiologic Studies Depression Scale [CES-D: (34)], consisting of 20 items scored on a 4-point scale with higher scores indicating more severe levels of depression was used. The internal reliability of Cronbach’s *α* = 0.93 was obtained in our study. Anxiety was measured with the State–Trait Anxiety Inventory-X-2 [STAI-X-2: (35)], composed of 20 items rated on a 4-point scale, with a higher score indicating higher levels of trait anxiety. We obtained Cronbach’s *α* = 0.91 in our study. Finally, subjective loneliness was measured twice using RULS v.3 in a 2-month interval, whereby Cronbach’s *α* = 0.95 and *α* = 0.93 were obtained, respectively. Only the participants with loneliness scores in the top and bottom quartiles in both measurement phases were asked to participate in the neurocognitive testing procedure. Those who agreed to participate were individually tested within 2 weeks using the neurocognitive functioning test battery, which consisted of and was sequenced as follows: (1) Block Design, (2) Auditory Verbal Learning Test [AVLT: (36)], (3) Rey-Osterrieth Complex Figure Drawing Test [ROCF: (37)], (4) Digit Span, (5) Trail Making Test-A and B [TMT-A, B: (38)], (6) Stroop Task (39), (7) Vocabulary, (8) Digit Symbol Coding Test, and (9) Wisconsin Card Sorting Test [WCST: (40)]. Among the test battery, the Block Design, Digit Span, Vocabulary, and Symbol Writing tests were taken from the Korean-Wechsler Intelligence Scale – fourth edition [K-WAIS-IV: (41)]. (For a detailed description of the NCFT battery and its normative data on the outcomes by groups, refer to Supplementary materials 1 and 2, respectively).

### Statistical analysis

First, descriptive statistics were carried out on the performance of each group (Table 1), and the normality of the outcome measures was examined (see Supplementary material 2). For the measures with an abnormal distribution according to the Shapiro–Wilk test (42), we applied the Mann–Whitney U-test. Otherwise, we applied the independent group t-test. Accordingly, we found the high-loneliness group to have significantly higher scores in depression (*t* = −4.63, *p* < 0.001) and anxiety (*t* = −4.56, *p* < 0.001) than their low-loneliness counterparts (see Table 1). Hence, we further examined the group differences in the normally distributed variables found to be significant by carrying out the analysis of covariance (ANCOVA) controlling for depression, anxiety, and both, respectively. For those that did not meet the assumption of normality, we carried out the non-parametric Quade’s ANCOVA (43). In addition, we carried out a *post-hoc* mediation analysis to examine the role of depression and anxiety in the association of loneliness and outcome variables that exhibited significant differences between the groups by using Hayes Process Macro (44) for normally distributed variables. As for the variables that did not meet the assumptions of normality, the robust bootstrap test ROBMED for mediation analysis was used (45) since it is less sensitive to deviations from model assumptions such as outliers and heavily tailed distributions. All statistical analyses were carried out with SPSS 28.0 (IBM SPSS, Armonk, NY).

## Results

### Group differences in neurocognitive functioning

Besides anxiety and depression, we found significant group differences in a number of neurocognitive measures. As shown in Table 1, the high-loneliness group showed poorer performance in the K-WAIS digit span forward trial compared with the low-loneliness counterpart (*t* = 2.42*), which is related to attentional functioning. The high-loneliness group also showed significantly more WCST perseveration responses (*t* = −2.32*), Stroop color/non-word trial errors (*U* = −2.69**), and color/word mismatch trial errors (*U* = −2.32*). These measures are largely associated with executive functioning.

### Group comparison of neurocognitive functioning using analysis of covariance and non-parametric Quade’s ANCOVA

For the significant variable whose assumption of normality was met (i.e., K-WAIS Digit Span Forward and WCST perseveration), we carried out an ANCOVA controlling for depression and anxiety on the neurocognitive variables. For non-normal measures (i.e., Stroop color-non-word error and color-word mismatch error), we applied Quade’s non-parametric ANCOVA (43). As shown in Table 2, when controlling for depression, WCST perseveration and Stroop color/non-word error maintained their statistical significance. Controlling for anxiety, all variables retained their statistical significance except for the Stroop color/word mismatch error. Finally, when both depression and anxiety were controlled as covariates, WCST perseveration and Stroop color/non-word error still maintained their statistical significance.

**Table 2:** Analysis of covariance (ANCOVA) or Quade’s non-parametric ANCOVA on neurocognitive measures controlling for depression and/or anxiety.

Covariate	Dependent variable	SS	df	MS	*F*	*p*	*η* ^2^
Depression	WCST perseveration	291.33	1	291.33	4.31*	*0.04*	0.08
	K-WAIS digit span forward	10.01	1	10.01	3.44	*0.07*	0.06
	STROOP color/non-word error	699.02	1	699.02	5.26*	*0.03*	0.09
	Color/word mismatch error	586.28	1	586.28	3.30	*0.08*	0.06
Anxiety	WCST perseveration	274.11	1	276.14	4.08*	*0.05*	0.07
	K-WAIS digit span forward	12.77	1	12.77	4.39*	*0.04*	0.08
	STROOP color/non-word error	734.16	1	734.16	5.56*	*0.02*	0.10
	Color/word mismatch error	562.88	1	562.88	3.19	*0.08*	0.06
Depression + Anxiety	WCST perseveration	274.11	1	274.11	3.98*	*0.05*	0.07
	K-WAIS digit span forward	10.14	1	10.14	3.42	*0.07*	0.06
	STROOP color/non-word error	673.20	1	673.20	5.07*	*0.03*	0.09
	Color/word mismatch error	537.75	1	537.75	3.04	*0.09*	0.06

*p < 0.05, K-WAIS, Korean-Wechsler Intelligence Scale; WCST, Wisconsin Card Sorting Test.

### Mediation effects of depression and/or anxiety between loneliness and neurocognitive functioning

As a result of conducting a *post-hoc* mediation analysis of depression and anxiety on the variables with significant group differences, we did not uncover any significant mediation effects of either variable on the relationships between loneliness and significant neurocognitive measures, respectively.

## Discussion

In this study, we examined whether loneliness may have significant implications on the mental functioning of a young population by using a comprehensive NCF test battery. In the initial analysis, the high-loneliness group showed more severe levels of depression and anxiety as well as poorer performance in measures related to executive functioning and attention, which was in line with previous findings on cognitive decline attributed to loneliness (7, 46). Even when controlling for depression and anxiety as covariates, the high-loneliness group showed significantly poorer performance in tasks related to executive functioning than their low-loneliness counterparts.

The neurocognitive variables that significantly differed between the high- and low-loneliness groups prior to controlling for depression and anxiety were K-WAIS Digit Span forward, WCST perseverative response, Stroop color-non-word error, and color/word mismatch error. These measures involve attentional functioning, which has also been reported to show deficits in depression (47).

The high-loneliness group showed significantly poorer performance in the WCST perseverative response and Stroop color-non-word error, even when depression and anxiety were controlled as covariates. The perseverative response in WCST reflects difficulty in set-shifting or an inability to recognize changes in the selection rule. The Stroop test, on the other hand, generally reflects accuracy in the processing of mismatching cues and controlled behavioral inhibition. The reason for the color/word mismatch error losing its statistical significance when controlled for depression and/or anxiety can be attributed to the limited sample and design of the study, besides the presence of their negative effects on performance. Our overall results suggest that young people high in loneliness may be more vulnerable to problems related to impulsive and addictive behaviors (48).

While we included measures of IQ, memory, and psychomotor functioning in our test battery, we did not obtain any significant group differences in these measures. Hence, it can be suggested that the negative impact of loneliness on cognitive functioning during early adulthood may begin with executive functioning and attention and then progressively spread to other domains of cognitive functioning, as reported in the older adult populations (6, 7). Future studies should apply more comprehensive measures of executive functioning and attention to various age groups to confirm our results.

In addition, we have controlled for depression and anxiety through the ANCOVA and carried out a separate mediational analysis on the effects of both variables on the association between loneliness and neurocognitive functioning, using non-parametric tests where appropriate. The results consistently confirmed that loneliness has a direct effect on the measures of executive functioning and attention, although the lack of a significant mediational effect of depression and anxiety should be confirmed in future research with a larger sample. Furthermore, studies to identify possible mediating variables between loneliness and neurocognitive functioning deficits may provide valuable implications for interventions to alleviate the negative effects of loneliness on cognitive functioning in the young adult population.

Finally, our study was one of the first investigations into the link between loneliness and cognitive functioning in a relatively young population using a comprehensive neurocognitive functioning test battery. Nonetheless, there are a few limitations of this preliminary study that should be considered when interpreting the results. First, our results should be confirmed using larger samples of different age groups and demographic backgrounds to ensure generalizability. Second, this study is cross-sectional in design, so caution should be taken when inferring causality between loneliness and neurocognitive functioning until further longitudinal studies have been conducted. Third, the measure of loneliness that we used is largely a subjective measure; hence, more objective measures of social isolation should be applied in future studies to validate our results. Finally, some measures in our battery may be overlapped and reflect more than one functional domain, e.g., the K-WAIS Digit Symbol Coding task may reflect both psychomotor speed and visual working memory. Future studies should aim to apply more refined measures of neurocognitive functioning to confirm and expand our results.

## Data availability statement

The raw data supporting the conclusions of this article will be made available by the authors, without undue reservation.

## Ethics statement

The studies involving humans were approved by the research Ethics Committee of Chonnam National University. The studies were conducted in accordance with the local legislation and institutional requirements. The participants provided their written informed consent to participate in this study. Written informed consent was obtained from the individual(s) for the publication of any potentially identifiable images or data included in this article.

## Author contributions

EJ: Data curation, Formal analysis, Investigation, Writing – original draft. SH: Conceptualization, Investigation, Methodology, Supervision, Validation, Writing – original draft, Writing – review and editing.
