# Supplementary material for: A preliminary study on the neurocognitive deficits associated with loneliness in young adults
Source: Front Public Health. 2024 Apr 12;12:1371063. doi: 10.3389/fpubh.2024.1371063 (PMC11046705; doi:10.3389/fpubh.2024.1371063)
Supplement: Supplementary file 1 [file Data_Sheet_1.doc]

**S1. Neuro-cognitive Function Test (NCFT) Battery**

**Neuro-cognitive Function Test (NCFT) Battery**

The measures contained in our NCFT battery cover many of the cognitive domains to be considered when assessing mental disorders according to DSM (APA, 2013), including complex attention, executive function, learning and memory, language, perceptual motor function, and social cognition. We attempted to construct a NCFT battery which included most domains covered in recent studies on the relationships between loneliness and neuro-cognitive functioning (Kang & Oremus, 2023; Tao, *et al.*, 2022; Estrella, *et al.*, 2021, 2023; Kyröläinen & Kuperman, 2021; Hajak, *et al.*, 2020).

*Intelligence(IQ)* was examined by obtaining Vocabulary and Block Design subtest scores of the K-WAIS-IV (Choe, *et al.*, 2014). Vocabulary has been considered to reflect general mental ability more than most other tests (Jensen, 2001), while Block Design measures fluid intelligence which involves spatial perception and visuo-motor coordination (Negri, *et al.*, 2022). The reliability of these two subtests reflecting general intelligence was *α* = .65 as presented in Table 1.

*Attention* included the Digit Span forward trial and Digit Symbol-Coding subtests of the K-WAIS-IV. *Memory* was measured with ROCF immediate and delayed drawing scores and AVLT immediate recall error and delayed recognition error. The ROCF has been widely used as a measure of visuo-constructional ability and non-verbal memory (Zhang, *et al.*, 2021), whereas AVLT assesses verbal learning and memory (Schmidt, 1996). We obtained internal reliability of *α* = .64, and *α* = .77, respectively.

*Executive function* was measured by the perseverative response of WCST (Grant & Berg, 2014) and number of errors in the Stroop (Stroop, 1935) subtests. The perseveration score in the WCST indicates the tendency to repeat the same kind of error even when the rule for the correct answer has shifted. The Stroop task involved reading a series of word list presented on the computer screen. Word error indicates the number of errors committed in reading color words printed in black letters. Color-Word error is the number of errors committed in reading color words printed in a matching color. Color-Nonword error indicates the number of errors committed when speaking out the printed color of the letters ‘XXXXX’. Color-Word Mismatch Error is speaking out a color other than the printed color of the color word, such as the printed word that differs from the printed color. In addition, Interference Error is derived by subtracting Word Error from Color-Word Mismatch Error. The overall internal consistency was Cronbach’s *α* = .70.

*Psychomotor functioning* was measured by using the error rates of TMT-A and B (Lezak, 2012; Cronbach’s *α* = .38). TMT-A involves connecting dotted numbers in order with a line (e.g., 1-2-3-4-5), whereas TMT-B involves connecting the numbers and alphabets in an alternating order (e.g., 1-A-2-B-3). TMT-A primarily reflects cognitive processing speed in motor control while TMT-B reflects mental shifting in executive functioning (Tombaugh, 2004).
