# Supplementary material for: A preliminary study on the neurocognitive deficits associated with loneliness in young adults
Source: Front Public Health. 2024 Apr 12;12:1371063. doi: 10.3389/fpubh.2024.1371063 (PMC11046705; doi:10.3389/fpubh.2024.1371063)
Supplement: Supplementary file 2 [file Table_1.doc]

**S2. Test of Normality of the NCFT Measures in Low- and High-Loneliness Groups**

| **Table 1. Test of Normality of the NCFT Measures in Low- and High-Loneliness Groups** | | | | | | | | |
| --- | --- | --- | --- | --- | --- | --- | --- | --- |
| **Variables** | | **Low-Loneliness** | |  |  | **High-Loneliness** | | |
|  | | Skewness | Kurtosis | Shapiro-Wilk | Skewness | | Kurtosis | Shapiro-Wilk |
| **IQ** | **K-WAIS**  Vocabulary | -0.36 | 0.55 | .99 | 1.00 | | 1.13 | .92 |
|  | Block Design | -1.85 | 3.76 | .78*** | -1.48 | | 1.54 | .85*** |
| **Attention** | **K-WAIS**  Digit Span Forward | -1.20 | 0.25 | .95 | -0.57 | | -0.94 | .96 |
|  | Digit Symbol-Coding | -1.69 | 7.30 | .85*** | -0.28 | | -0.60 | .96 |
| **Memory** | **ROCF**  Copy | -1.43 | 0.91 | .72*** | -1.60 | | 1.75 | .65*** |
|  | Immediate | -1.31 | 2.02 | .89*** | -0.17 | | -0.40 | .97 |
|  | Delayed | -1.12 | 1.20 | .87*** | -0.46 | | -0.16 | .97 |
|  | **AVLT**  Immediate Recall Error | -0.52 | -0.70 | .31*** | -0.33 | | -1.02 | .56*** |
|  | Delayed Recognition Error | 2.43 | 4.17 | .39*** | 2.83 | | 7.92 | .42*** |
| **Executive Function** | **WCST**  Perseveration | 1.73 | 3.75 | .94 | 0.86 | | -0.08 | .93 |
|  | **STROOP**  Word Error | 3.86 | 13.74 | .27*** | 1.70 | | 0.98 | .47*** |
|  | Color-Word Error | 2.93 | 7.78 | .40*** | 2.32 | | 5.06 | .50*** |
|  | Color-Nonword Error | 3.11 | 9.82 | .40*** | 0.77 | | -0.65 | .74*** |
|  | Color-Word Mismatch Error | 2.27 | 5.70 | .59*** | 0.44 | | -1.21 | .81*** |
|  | Interference Error | 1.99 | 5.25 | .65** | 0.16 | | -0.86 | .90* |
| **Psycho-Motor** | **TMT**  A Trial Error | 2.98 | 7.34 | .33*** | - | |  | - |
|  | B Trial Error | 3.11 | 9.82 | .40*** | 2.32 | | 5.06 | .50*** |

* *p* < .05, ** *p* < .01, *** *p* < .001

AVLT = Auditory Verbal Learning Test, IQ = General Intelligence, K-WAIS = Korean-Wechsler Intelligence Scale, ROCF = Rey-Osterrieth Complex Figure Drawing Test, TMT = Trail Making Test, WCST = Wisconsin Card
